# Access and reattachment of biliary tree anomaly through Roux-en-Y hepaticojejunostomy: A case report

**DOI:** 10.1016/j.radcr.2024.04.068

**Published:** 2024-05-22

**Authors:** Caroline J. Cushman, Andrew F. Ibrahim, Jack Rostas, James Montgomery

**Affiliations:** a School of Medicine, Texas Tech University Health Sciences Center, Lubbock, TX, USA; b Department of Surgery, Covenant Medical Center, Lubbock, TX, USA; c Department of Interventional Radiology, Covenant Medical Center, Lubbock, TX, USA

**Keywords:** Roux-en-Y hepaticojejunostomy, Biliary tree anomaly, Cholecystectomy, Case report

## Abstract

The right posterior segmental duct (RPSD) draining into the cystic duct is exceedingly rare. Ligation of the cystic duct in proximity to the junction of an aberrant right hepatic duct after a cholecystectomy can lead to life threatening complications. The present case study reveals a severed anomalous RPSD and subsequent Roux-en-Y hepaticojejunostomy procedure employed to fix biliary anomaly.

## Introduction

Congenital variations in bile duct branching patterns represent one of the major causes of iatrogenic bile duct injury (BDI) during laparoscopic cholecystectomy and remain one of the most devastating complications [1]. They usually occur as a consequence in misidentification of the biliary tree due to anomalous branching patterns [2]. The most classified variation of bile duct branching, in 27.6% of subjects involves the right posterior sectoral duct (RPSD) and its joining with the left hepatic duct (LHD) [3]. However, the anomalous drainage of the RPSD into the cystic duct is extremely rare and is estimated to occur in 0.8% [3]. In Choi's classification system of right hepatic duct anomalies this is considered Type 3C [4]. This exceptional anomaly signifies an exceedingly uncommon and perilous bile duct arrangement, making it highly susceptible to injury during cholecystectomy. Biliary tree anomalies pose a genuine surgical challenge during routine procedures like laparoscopic cholecystectomy (LC) and may lead to severe complications due to inadvertent ligation.

## Case presentation

**Fig. 1:**
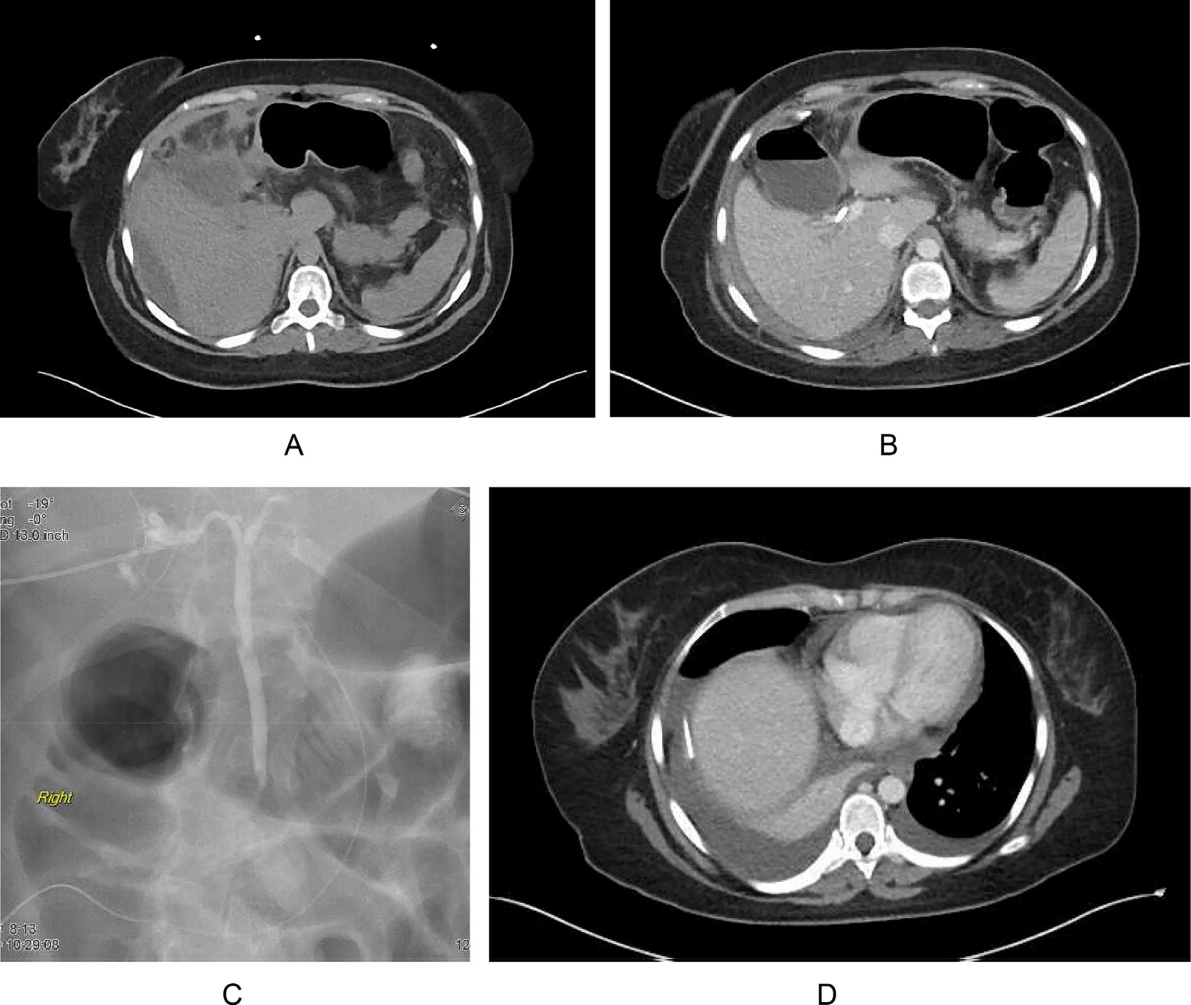
(A) Accumulation of biliary fluid in the gallbladder fossa and ascites. (B) Intra-abdominal infected subhepatic biloma with air fluid level. (C) Percutaneous transhepatic cholangiography (PTC) to place a biliary stent in the right anterior segmental duct. PTC placement of biliary drain confirmed leak was coming from elsewhere in biliary system. (D) Development of hydropneumothorax.

**Fig. 2:**
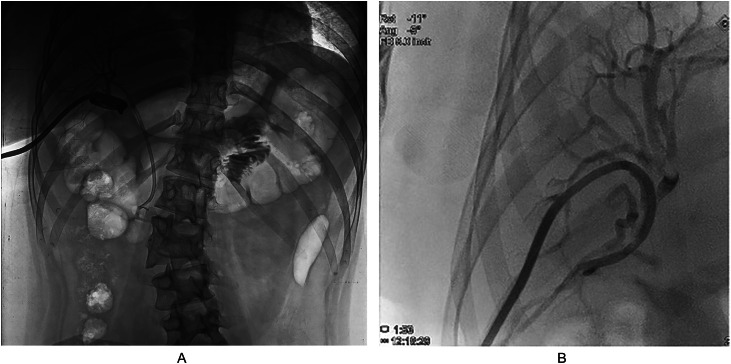
(A) Fluoroscopy-assisted abscessogram in the right subhepatic space confirms communication with right posterior segmental biliary duct (RPSD). (B) Percutaneous transhepatic cholangiography (PTC) with tube placement in right posterior segmental duct (RPSD) for Roux-en-Y hepaticojejunostomy procedure.

A 29-year-old woman with a medical history of constipation, scoliosis, and alcoholism arrives at the emergency department (ED), complaining of epigastric abdominal pain that radiates to the back. Additionally, she reports symptoms such as nausea, vomiting, nonbloody nonbilious emesis, and postprandial pain. On physical examination, she has tachycardia and hypertension. One month later, during a follow-up examination, ultrasonography revealed the presence of a gallbladder calculus with a thickened wall and trabeculated mucosa consistent with acute cholecystitis. Subsequently, a cholecystectomy procedure was conducted and deemed successful. However, persistent abdominal pain and distention occurred. Following this, a subsequent computed tomography (CT) scan showed the existence of ascites and the accumulation of fluid in the gallbladder fossa (Fig. 1A). Two weeks after undergoing laparoscopic cholecystectomy, the patient arrived at the emergency department with symptoms of septic shock. The admission diagnosis indicated the presence of retained intra-abdominal infected subhepatic biloma with air fluid level, moderate ascites, and a probable bile duct leak (Fig. 1B). CT guided right upper quadrant abdominal drain placement and CT guided aspiration of left lower quadrant fluid collection were performed. The microbiology report indicated the presence of Streptococcus Viridians and Candida glabrata in the fluid collection. The pigtail drains with suction revealed 200 mL of bile collection per day. Additionally, attempted endoscopic retrograde cholangiopancreatography (ERCP) during hospitalization that failed due to anatomical difficulty. At this time, it was suspected that there was a surgical complication that led to a leak in the common bile duct. The patient underwent ultrasound-guided percutaneous transhepatic cholangiography (PTC) to place a biliary stent in the right anterior hepatic duct (Fig. 1C). Unfortunately, the interventional techniques failed in resolving the leak as right subhepatic and a left lower quadrant biloma collections were persistent. During a follow-up clinic appointment, it was discovered that the patient had developed a hydropneumothorax, leading to the sequential placement of a chest tube (Fig. 1D). Due to continued biliary leak after stent placement there was a suspected anomalous duct. Afterward, a fluoroscopy-assisted abscessogram was conducted by injecting contrast material into the biloma drain. The procedure confirmed the severance of an aberrant RPSD directly attached to the cystic duct from the prior cholecystectomy procedure, as evidenced by contrast extending into the RPSD (Fig. 2A). Shortly after, access to the RPSD was obtained by interventional radiology, accompanied by the placement of a biliary drain to facilitate access for the Roux-en-Y hepaticojejunostomy procedure (Fig. 2B). The successful attachment of the anomalous right hepatic duct to the proximal jejunum was achieved. A follow-up cholangiogram was performed, revealing clear visualization of the biliary ducts, with the contrast extending into the small bowel. The patient currently possesses a surgically constructed double common bile duct (DCBD), and subsequent follow-up examinations have revealed no troubling complications.

## Discussion

In laparoscopic cholecystectomy procedures common risk factors include arterial injuries, gallbladder adhesions, and anomalous bile duct variations [2]. The occurrence of bile duct injury, considered one of the most serious complications of laparoscopic cholecystectomy (LC), has been documented to occur 3% [5]. As seen in this patient bile duct injuries cause significant short- and long-term morbidity including major operations, multiple hospitalizations, biliary strictures, peritonitis, sepsis, and biloma formation [2,[6], [7], [8], [9]]. Earlier findings indicated that cases with aberrant bile ducts had a higher occurrence of bile duct injury compared to cases without such anomalies [10]. However, the incidence of a Type 3C is exceedingly anomalous in comparison to other biliary tree configurations [4]. Moreover, performing a PTC in a nondilated system is challenging, and dealing with only a small segment (RPSD) adds further complexity and difficulty to the procedure. PTC access was essential for reconstruction, as the small RPSD is not distinctly visible without a catheter, ensuring location for bowel sutures.

Early acknowledgement and recognition will assist in prevention of biliary duct ligation. Post-operative imaging such as magnetic resonance cholangiopancreatography (MRCP) was performed in this patient but was inconclusive. However, this is the gold standard for least invasive visualization for biliary anomalies and is especially important in being done preoperatively for diagnoses of a biliary anomaly [11]. Hence, considering the rarity of this anomaly, alternative imaging modalities like drip infusion cholecystocholangiography computed tomography (DIC-CT) may be contemplated [2]. A study demonstrated the potential of identifying an aberrant RPSD using DIC-CT. This served as an alternative, as confirmation was challenging with MRCP or endoscopic retrograde cholangiopancreatography (ERCP) [12]. According to findings, DIC-CT exhibited clear identification of both aberrant bile ducts and cystic ducts in contrast to MRCP [13]. Consequently, considering the enhanced clarity offered by DIC-CT, it may prove beneficial to conduct this imaging modality when the suspicion of an aberrant bile duct arises based on MRCP results [2]. Another imaging modality, Intraoperative cholangiogram (IOC) has the potential to enhance the detection of abnormalities in the common bile duct, increasing the likelihood of recognition in BDI's but would not have worked for a Type 3C ligation variance [4].

ERCP, an interventional technique, plays a central role in diagnosis of BDI, however correct identification can only be achieved in less than 20% of patients [14,15]. The diagnostic limitations of ERCP derive from the lack of intercommunication between transected sectoral or hepatic duct and common bile duct. In this case presentation, ERCP failed to delineate cystohepatic ducts when they have been transected and has no role in management of Type 3C. However, ERCP indirectly verified that there was a hepatic duct injury, as evidenced by the persistent leak even after intervention.

Two additional primary interventional procedures are available for treating post-surgical biliary injuries: PTC with either external or internal stent insertion. Typically, PTC with external stent placement is conducted on the right anterior segmental duct or the left bile duct due to anatomical convenience. However, this procedure was slightly adjusted to accommodate the anomalous duct, leading to PTC with stent placement performed on the RPSD. An alternative approach was PTC with internal stent insertion through ERCP, but as previously mentioned, this was not anatomically feasible due to the location of the ligated anomalous duct.

Many cases with biliary leaks involve the development of papillary stenosis, necessitating the initial correction of this condition before proceeding further. In cases of papillary stenosis, it's advisable to perform balloon dilatation of the common bile duct stricture using PTC before addressing the biliary leak. This approach helps relieve pressure and prevent further bile accumulation in the subhepatic space.

If the Roux-en-Y hepaticojejunostomy procedure for the patient had not been successful, a right lobe hepatectomy would have been necessary. This alternative procedure would have entailed a challenging recovery process and posed additional risks, including but not limited to bleeding, infection, impaired liver function, and more.

**Fig. 3:**
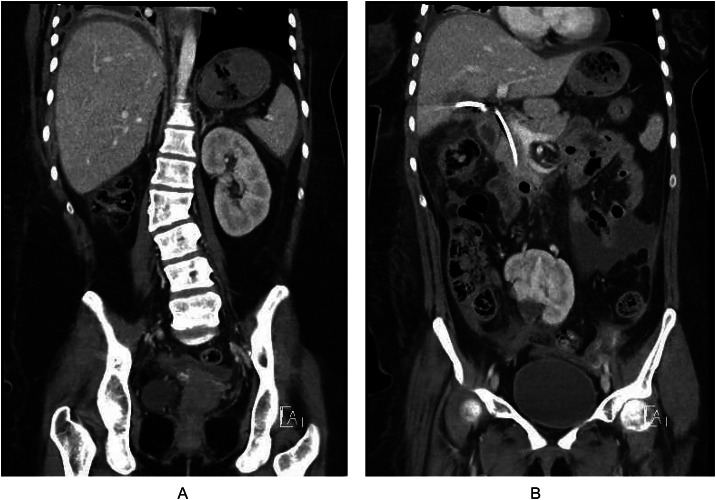
(A) Congenital L2 hemivertebrae with fusion to L1. (B) Right pelvic kidney.

Additionally, it should be noted this patient had 2 other congenital anomalies, a pelvic kidney and L2 hemivertebrae with fusion to L1 (Figs. 3A and B). To our knowledge this patient has not had genetic testing. However, genetic syndromes such as Alagille syndrome and Brens syndrome are associated with anomalous malformations in skeletal vertebrae, biliary tree, and kidneys [16,17].

## Conclusion

Conducting preoperative and intraoperative evaluations of the biliary duct and mindfulness of variations in aberrant biliary ducts are essential preventive measures to avoid bile duct injuries. Thereby avoiding early biliary leaks, peritonitis, and sepsis [1]. Employment of interventional radiology guided Roux-en-Y hepaticojejunostomy procedure provided a definitive surgical solution for the biliary tree anomaly. Given the limited number of clinical cases involving this anomaly, treatment protocols remain unclear. Conducting future clinical research would be beneficial to assess the risks and outcomes, particularly in the long term, associated with the use of the Roux-en-Y hepaticojejunostomy procedure for surgically created double common bile ducts (DCBD) of RPSD.

## Patient consent

Written informed consent for the publication of this case report was obtained from the patient.
